# Heat Treatment-Assisted Optimization of the Water Splitting Performance of CoCrNi0.5Ti0.3V0.2Al0.4 Eutectic High-Entropy Alloy

**DOI:** 10.3390/ma18174015

**Published:** 2025-08-27

**Authors:** Mingran Sun, Zixiang Yin, Shuai Liu, Yangchuan Cai, Yu Zhang

**Affiliations:** 1 School of Materials Science and Engineering, Tianjin University of Technology, Tianjin 300384, China; m15081643686@163.com (M.S.); 15290259270@163.com (Z.Y.); 15933687828@163.com (S.L.); 2 Tianjin Key Laboratory of Advanced Functional Porous Materials, Institute for New Energy Materials and Low-Carbon Technologies, School of Materials Science and Engineering, Tianjin University of Technology, Tianjin 300384, China

**Keywords:** eutectic high-entropy alloy, nanoporous materials, heat treatment, dealloying, microstructure, electrocatalytic performance

## Abstract

In this study, the synergistic tuning mechanism of heat treatment (600, 800, and 1000 °C) and dealloying (40, 60, and 80 °C) on the microstructure and electrocatalytic performance of an FCC + BCC-type CoCrNi0.5Ti0.3V0.2Al0.4 eutectic high-entropy alloy (EHEA) was systematically investigated. The findings indicate that with an increase in heat treatment temperature, there is a gradual increase in grain size and a change in the fraction of the two phases. Notably, heat treatment at 800 °C resulted in an FCC-dominated dual-phase structure with uniformly refined grains. As the dealloying temperature increased, the pore size also increased, leading to a uniform distribution of the internal FCC and BCC phases. The sample subjected to heat treatment at 800 °C and dealloying at 80 °C exhibited an OER overpotential of only 265 mV and a Tafel slope of 67.84 mV/dec, significantly enhancing the electrocatalytic activity and stability of the alloy. This study elucidates the mechanism by which the combination of heat treatment and dealloying processes optimizes the electrocatalytic performance of eutectic high-entropy alloys, providing a novel strategy for the design of non-precious metal electrocatalysts.

## 1. Introduction

Water electrolysis is considered a key technology for developing clean energy and consists of two half-reactions: the anodic oxygen evolution reaction (OER) and the cathodic hydrogen evolution reaction (HER) [1]. However, the kinetics of this reaction are typically slow [2], necessitating the use of high-performance electrochemical catalysts to improve efficiency. Pt and its alloys are regarded as the best HER electrocatalysts, whereas IrO_2_ and RuO_2_ are generally considered the best OER electrocatalysts [3,4]. However, their high cost and low elemental abundance preclude them from meeting the requirements of large-scale practical applications. Therefore, researchers have begun exploring water-splitting catalysts that are low-cost, highly active, and have high energy conversion efficiency.

Nanoporous metals have been extensively studied because of their high specific surface area, good electrical and thermal conductivity, catalyst recyclability, and high mechanical rigidity [5,6,7]. Forty et al. [8] were the first to study the dealloying of an Au–Ag alloy in concentrated nitric acid to form nanoporous gold and proposed a pore formation mechanism. Chen et al. [9] dealloyed a Cu_19_Ti_1_Al_80_ precursor alloy to obtain a bimodal nanoporous CuTi (np-CuTi), which achieved a specific surface area of 46 m^2^·g^−1^ and exhibited a HER onset overpotential comparable to that of commercial Pt/C catalysts along with a higher current density in 0.1 M KOH. Zhang et al. [10] dealloyed a Ni_2_Fe_1_Al_97_ precursor (composed of α-Al, Al_3_Ni, and Al_13_Fe_4_ phases) using 1 M NaOH, yielding a bimodal porous nanowire network consisting of NiFe_2_O_4_ and Ni phases. In 1 M KOH, this catalyst delivered an OER overpotential of 244 mV at 10 mA·cm^−2^ with a Tafel slope of 39 mV·dec^−1^. These studies demonstrate that nanoporous metals prepared by dealloying possess a self-supporting three-dimensional porous structure. The continuous metallic ligament network not only provides electron transport pathways during electrocatalysis, but its large specific surface area ensures that active sites on the ligament surfaces or any species loaded on the ligaments are fully exposed, thereby increasing the utilization of catalytically active sites. Meanwhile, the interconnected pore channels ensure sufficient diffusion of the reactants and products through the electrolyte during electrocatalysis.

At present, traditional alloy catalysts for water splitting have a limited compositional tuning range, which restricts the modulation of the electronic structure and chemical adsorption of reaction intermediates [11]. High-entropy alloys (HEAs), first proposed by Yeh [12] and Cantor [13], are a new class of alloys consisting of five or more principal elements in equiatomic or near-equiatomic ratios [14,15]. Owing to their lattice distortion effect, high-entropy effect, cocktail effect, and sluggish diffusion effect, HEAs have potential applications in electrocatalysis [16,17,18]. The disparity in atomic sizes induces significant lattice distortion, which helps reduce the overall energy of the system and facilitates the activation of active sites as well as the transport of active species during catalysis [19,20]. The high-entropy effect promotes the formation of a single solid-solution phase and suppresses the formation of intermetallic compounds. The high configurational entropy and low Gibbs free energy confer exceptional thermal and chemical stability to the crystal structures. The presence of multiple elements in the same lattice, that is, multiple types of atomic sites, significantly expands the tunability of the catalytically active sites for electrocatalytic reactions [21]. The cocktail effect results in a disordered surface that increases the variety of active sites with different adsorption behaviors; these active sites can serve as reaction intermediates in electrocatalysis, yielding a performance superior to the simple sum of each component’s performance [22,23]. The sluggish diffusion effect facilitates the formation of fine precipitates, promotes the development of an amorphous structure, suppresses grain growth, and raises the recrystallization temperature, which effectively slows coarsening and enhances creep resistance, thereby controlling the formation of nanostructures [24].

Lu et al. [25] were the first to combine the concepts of eutectic alloys and high-entropy alloys, proposing the concept of eutectic high-entropy alloys (EHEAs). EHEAs possess the characteristics of both high-entropy and eutectic alloys, can form a dual-phase eutectic structure, and can achieve outstanding synergistic properties through compositional design. Eutectic microstructures can be used to fabricate electrode materials with complex hierarchical pore structures, which provide a greater specific surface area and more effective mass transfer channels, thereby improving the efficiency of water-splitting reactions [26,27]. Chen et al. [28] employed a phosphorus-doped Ni_30_Co_30_Fe_10_Cr_10_Al_18_W_2_ eutectic high-entropy alloy, finding that the 1P-HEA required overpotentials of only 70 mV and 211 mV to reach 10 mA·cm^−2^ for the hydrogen evolution reaction (HER) and oxygen evolution reaction (OER), respectively. Li et al. [29] used physical metallurgy techniques to alloy Zr with an FeCoNiCr medium-entropy alloy (MEA), thereby designing an FeCoNiCrZr_0_._25_ HEA with a multiphase spatial structure. This self-supporting catalyst features an interconnected tri-scale gradient porous architecture, achieving OER overpotentials of 215 mV at 10 mA·cm^−2^ and 370 mV at 1000 mA·cm^−2^. Yao et al. [30] developed a three-dimensional hierarchical nanoporous CuAlNiMoFe hybrid electrode that achieved current densities of 1840 mA·cm^−2^ in 1 M KOH and 100 mA·cm^−2^ in 1 M PBS at overpotentials of only 240 mV and 183 mV, respectively. Therefore, through rational composition design to form a eutectic microstructure, an excellent overall performance can be achieved.

Heat treatment is a common approach for tuning the microstructure of EHEAs and improving their properties [31,32,33]. Li et al. [34] heat-treated an Al_0.4_Co_0.5_V_0.6_FeNi eutectic high-entropy alloy at 1000, 1100, and 1200 °C and found that heat treatment at 1200 °C increased the FCC phase content, produced a denser microstructure, and led to pronounced compositional segregation. Sun et al. [35] studied the microstructure evolution and mechanical properties of an Al_0.9_Cr_0.8_FeMn_0.8_Ni_2.0_ EHEA annealed between 300 °C and 800 °C and found that annealing at 600 °C for 2 h significantly increased the BCC phase fraction, improving the alloy’s strength; at higher annealing temperatures (700–800 °C), a large amount of the FCC phase dissolved, leading to the formation of acicular ordered B2 (Al- and Ni-rich) precipitates and a reduction in mechanical properties. Zhang et al. [36] reported that heat treatment can optimize the strength of a Ni_20_Fe_20_Co_35_Cr_15_Mo_10_ HEA and adjust its HER efficiency; notably, the alloy heat-treated at 1150 °C exhibited the lowest HER overpotential. These studies indicate that appropriate heat treatment can optimize the microstructure and elemental distribution of EHEAs, thereby influencing the dealloying process and the resulting porous structure for water-splitting performance. However, existing studies on the effect of heat treatment on EHEAs have mainly focused on microstructural evolution, conventional mechanical properties, and corrosion properties, with few reports on its influence on the electrocatalytic performance for water splitting. Therefore, in this study, we employed heat treatment to optimize the microstructure of an EHEA and applied a suitable dealloying process to investigate their combined effects on water-splitting performance.

In summary, our team previously obtained an FCC + BCC-type CoCrNi0.5Ti0.3V0.2Al0.4 EHEA through phase diagram calculations. By utilizing phase-diagram-guided methods to control the relationship between heat treatment and microstructure and by pairing with an appropriate dealloying process, we aim to elucidate the coupling among heat treatment, microstructure, and water electrolysis performance, thereby providing theoretical support for the design of efficient electrocatalysts.

## 2. Materials and Methods

### 2.1. Alloy Preparation and Heat Treatment Process

In a previous study, our team used the Thermal-Calc thermodynamic software (Copyright © Thermo-Calc Software AB, Stockholm, Sweden) and Scheil model to simulate solidification and, based on the lever rule, designed a CoCrNi0.5Ti0.3V0.2Al0.4 eutectic high-entropy alloy [37]. This alloy was prepared from high-purity (>99.9 wt.%) Co, Cr, Ni, Ti, V, and Al and cast under an argon atmosphere using an XC-500 vacuum arc melting furnace (Shenyang Scientific Instrument Co., Ltd. Chinese Academy of Sciences, Shenyang, China). The ingot was remelted five times to ensure a uniform elemental distribution. The cast precursor was placed in a KSL-1200X muffle furnace (Hefei Kejing Materials Technology Co., Ltd., Hefei, China) and heated at 10 °C/min to the target temperatures (600, 800, and 1000 °C), held for 2 h, and then furnace-cooled to room temperature.

### 2.2. Dealloying Process

The cast precursor was cut into cuboids measuring 18 mm × 5 mm × 1 mm using electric discharge wire cutting. The specimens were immersed in 1 M HCl at 40, 60, and 80 °C for 1 h for dealloying, followed by rinsing with deionized water to identify the optimal dealloying conditions.

The precursor was then cut into cuboids of 18 mm × 5 mm × 0.5 mm. These samples were immersed in 1 M HCl under the selected optimal dealloying conditions to produce a porous structure, which was used as the water electrolysis electrode.

### 2.3. Material Characterization

The precursors before and after heat treatment were ground with abrasive paper, mechanically polished, and etched for 10 s with Kroll’s reagent (2% HF, 6% HNO_3_, 92% H_2_O). A Hitachi S-4700 scanning electron microscope (SEM) was used to characterize the microstructure, and an energy-dispersive X-ray spectroscope (EDS) was used to evaluate the elemental composition and its distribution. A Tecnai G2 F20 transmission electron microscope (TEM) was used to obtain high-resolution images, elemental maps, and selected area electron diffraction (SAED) patterns of the as-cast sample. The phase composition was analyzed using a Shimadzu XRD-6100 X-ray diffractometer (Shimadzu Corporation, Kyoto, Japan, Cu Kα radiation, 40 kV, 40 mA, scan speed 10°/min, 2θ range 20–100°). For the dealloyed samples, SEM was used to characterize the surface and cross-sectional morphologies, and XRD was used to analyze the phase composition. EDS was used to assess the elemental composition and distribution of the sample dealloyed at 60 °C.

### 2.4. Electrochemical Testing

Electrochemical measurements were conducted using a CHI 760E electrochemical workstation (CH Instruments, Inc., Austin, TX, USA) in a three-electrode configuration, with the sample as the working electrode, Hg/HgO as the reference electrode, and a carbon rod as the counter electrode. The electrolyte was a 1 M KOH solution. The tests included linear sweep voltammetry (LSV, scan rate 5 mV/s), cyclic voltammetry (CV, scan rates 20–100 mV/s), electrochemical impedance spectroscopy (EIS, frequency range 10^−2^–10^5^ Hz), and chronopotentiometric stability testing (48 h at a constant potential). All electrochemical tests were conducted in three independent replicate experiments to ensure statistical reliability of the results.

After the 48 h stability test, the sample was analyzed by XRD to determine the phase composition, by SEM to examine the surface and cross-sectional morphology, and by EDS to evaluate the elemental composition and distribution.

## 3. Results and Discussion

### 3.1. Effect of Heat Treatment on the Microstructure of the As-Cast CoCrNi0.5Ti0.3V0.2Al0.4 EHEA

**Figure 1:**
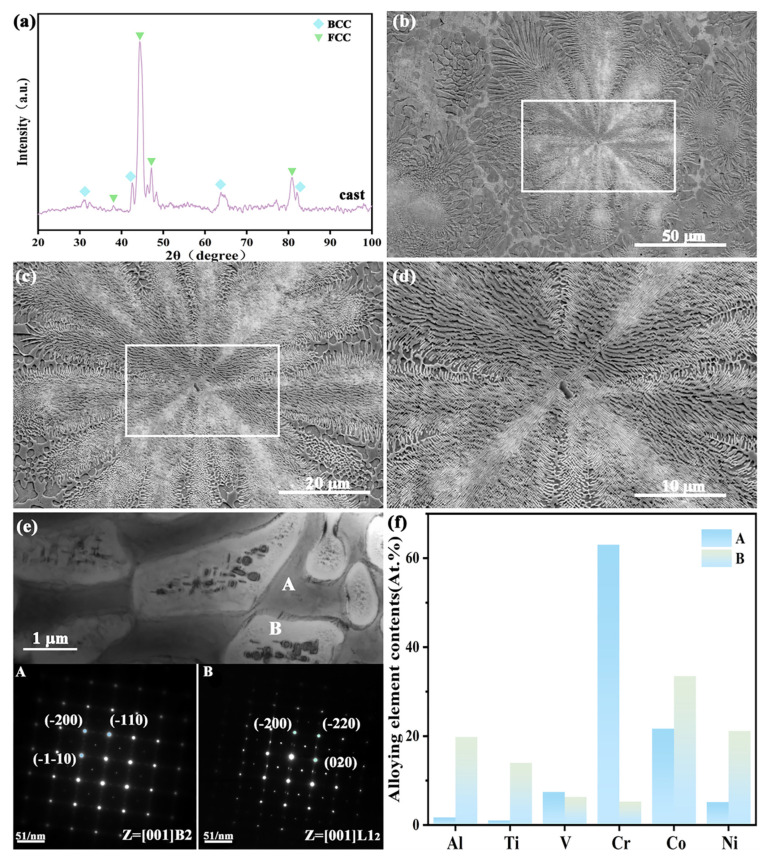
Phase composition, microstructure, and elemental distribution of the as-cast CoCrNi0.5Ti0.3V0.2Al0.4 EHEA: (**a**) XRD pattern; (**b**–**d**) SEM micrographs; (**e**) TEM image and SAED pattern; (**f**) elemental distribution.

XRD analysis (Figure 1a) indicates that the as-cast CoCrNi0.5Ti0.3V0.2Al0.4 EHEA is composed primarily of BCC and FCC phases, with an FCC characteristic peak at 47.28°. The SEM images (Figure 1b–d) show that the grains of the cast alloy exhibit a typical irregular eutectic morphology. Within the grains, the eutectic structure is very fine (100 nm lamellae thickness), whereas, near the grain boundaries, the eutectic structure is noticeably coarser (500 nm thick). The irregular eutectic morphology of this alloy mainly originates from the Ti-induced asymmetric growth mechanism. Ti preferentially segregates to the hard phase, leading to interfacial energy anisotropy and a kinetic imbalance between the FCC matrix and hard phase, which destabilizes the advancing eutectic front and results in a degenerate lamellar structure [38]. Meanwhile, Ti reduces the diffusivity of the melt, disrupting the coupled solute redistribution required for regular eutectic growth. The coarsening of the eutectic near the grain boundaries was attributed to a kinetic transition in the late stages of solidification. In the early stage of EHEA solidification, under a high cooling rate, the eutectic nucleates at a large undercooling [39]. In the final stage of solidification, solute enrichment in the grain boundary regions reduces the local undercooling, slowing the growth rate into a diffusion-controlled regime and significantly increasing the eutectic spacing [40]. Simultaneously, Ostwald ripening in the interdendritic liquid causes small eutectic colonies to dissolve, further exacerbating coarsening [41]. TEM examination of the fine irregular eutectic structure within the grains showed that the lighter regions were the FCC phase and the darker regions were the BCC phase. EDS results indicated that the FCC phase was enriched with Co and Ni, whereas the BCC phase was enriched with Cr. The enrichment of Cr in this phase can effectively enhance its corrosion resistance [42], so this phase was retained during the metallographic preparation, which differs from the microstructures reported in other FCC/BCC dual-phase eutectic HEAs.

**Figure 2:**
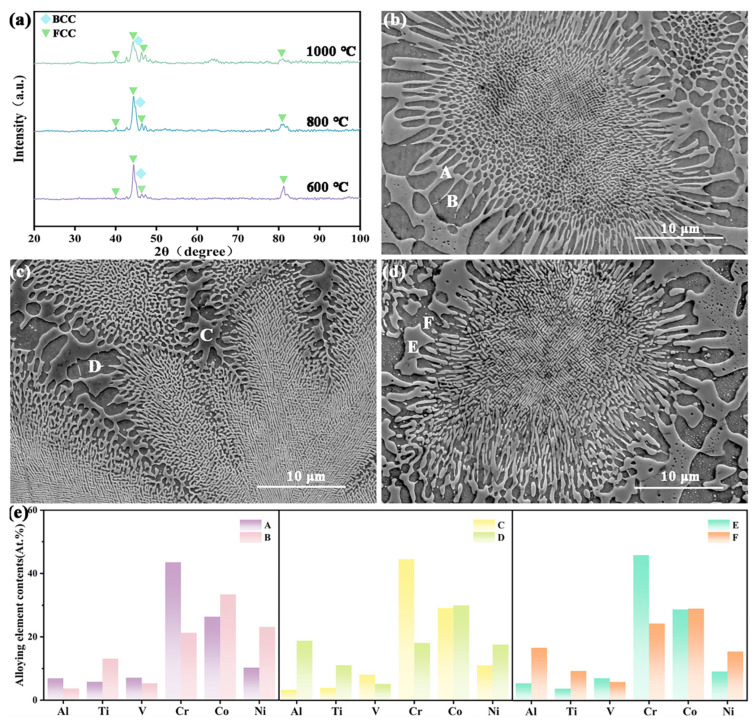
Phase constitution, microstructure, and elemental distribution of the CoCrNi0.5Ti0.3V0.2Al0.4 EHEA precursor after heat treatment: (**a**) XRD pattern; (**b**–**d**) SEM micrographs of (**b**) 600 °C sample, (**c**) 800 °C sample, and (**d**) 1000 °C sample; (**e**) elemental distribution.

XRD analysis of the heat-treated samples (Figure 2a) shows that for the HT-600 °C sample, the main peak of the FCC phase is at 44.42° with an intensity of 468.43. As the heat treatment temperature increased, the FCC peak first increased and then decreased, and the peak broadened. At 800 °C, the FCC main peak reached a maximum intensity of 475.22, which then weakened to 299.12 at 1000 °C, and the FCC peak gradually shifted to the left (to 44.28°) with increasing temperature. This suggests that 800 °C is the temperature at which FCC precipitation is most pronounced; element diffusion becomes active in this temperature range but has not yet reached full homogenization, resulting in significant compositional inhomogeneity within the FCC phase. Excessive heating causes over-diffusion of elements, leading to the dissolution of the FCC phase and a partial transformation to BCC. The newly formed BCC phase has very fine grains in the early stages of nucleation and growth with a high density of defects [43], causing peak broadening and accompanying lattice expansion. The SEM images (Figure 2b–d) show that with increasing heat treatment temperature, the phase constitution remains BCC + FCC, the grain size increases from 150 nm to 200 nm, the grain interiors become denser and more uniform, the microstructure near the grain boundaries becomes noticeably coarser, and the eutectic regions do not undergo phase decomposition, indicating that the eutectic structure has good thermal stability. EDS analysis indicated that the volume fraction of the FCC phase increased markedly at 800 °C, which was attributed to the enhanced diffusion rate of Co, resulting in the precipitation, growth, and coarsening of the FCC phase. Combining the SEM and XRD results, it is clear that heat treatment influences atomic diffusion, thereby altering the two-phase distribution and grain morphology.

### 3.2. Effect of Dealloying on the Porous Structure Formatting of Mathematical Components

**Figure 3:**
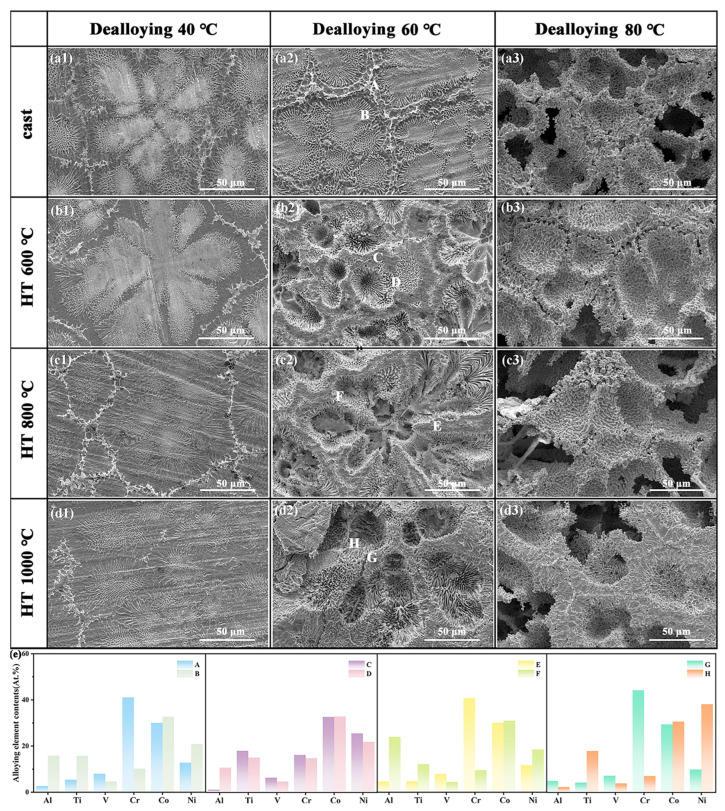
Surface microstructure and elemental distribution of CoCrNi0.5Ti0.3V0.2Al0.4 EHEA after heat treatment and subsequent dealloying: (**a**–**d**) SEM images; (**a1**–**d1**) dealloyed at 40 °C; (**a2**–**d2**) dealloyed at 60 °C; (**a3**–**d3**) dealloyed at 80 °C; (**e**) elemental distribution.

**Figure 4:**
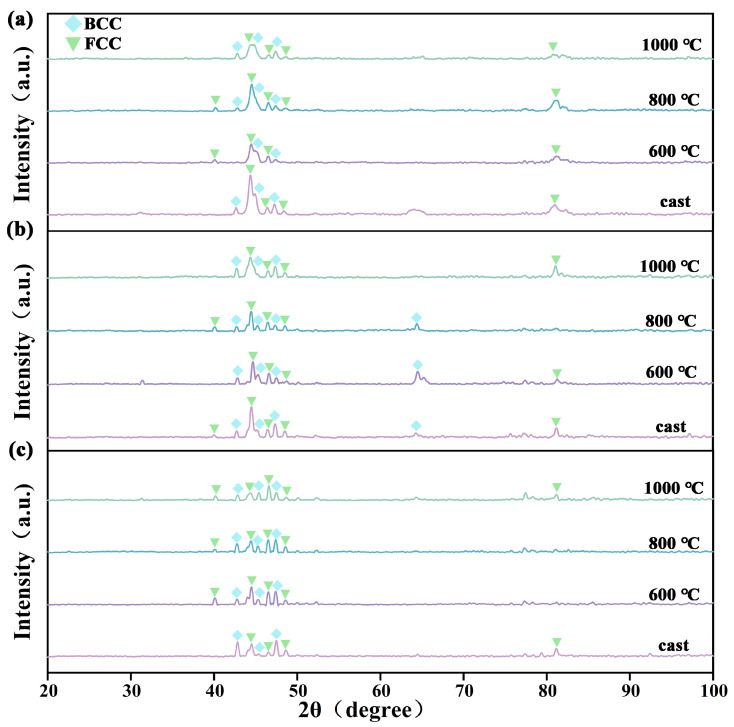
XRD patterns of CoCrNi0.5Ti0.3V0.2Al0.4 EHEA before and after heat treatment followed by dealloying: (**a**) dealloyed at 40 °C, (**b**) dealloyed at 60 °C, and (**c**) dealloyed at 80 °C.

Figure 3 shows the surface SEM images of the CoCrNi0.5Ti0.3V0.2Al0.4 EHEA precursor dealloyed at different temperatures. Upon dealloying at 40 °C (Figure 3(a1–d1)), the surface microstructure was similar to that of the precursor alloy, with a clearly visible eutectic structure and grain boundaries. At 60 °C (Figure 3(a2–d2)), the morphology of the as-cast sample after dealloying was similar to that at 40 °C, but the differences between the grain boundary regions and the grain interior became more pronounced. As the heat treatment temperature increased, the grain interior regions after dealloying became severely corroded, making the eutectic structure more distinct, whereas the grain boundary regions remained intact. When the dealloying temperature is raised to 80 °C (Figure 3(a3–d3)), the pore size increases to about 50–100 μm with a more uniform distribution, forming a regular honeycomb-like arrangement; the grain boundary regions are preserved, whereas the eutectic structure inside the grains is completely dissolved by the dealloying solution. EDS analysis (Figure 4) indicates that these observations are due to the unique eutectic dual-phase (FCC/BCC) structure and the markedly different electrochemical behaviors of these phases in a corrosive environment. The grain interior is primarily composed of a Co-rich FCC phase, which has a lower corrosion potential and tends to dissolve. In contrast, the grain boundary region is composed of a Cr-rich BCC phase, which, being rich in passivating elements, can form a highly stable protective oxide film and thus exhibits a higher corrosion potential and excellent corrosion resistance [44].

However, varying the heat treatment temperature also affects the surface morphology after dealloying. At lower heat treatment temperatures, atomic diffusion is limited, and the grain size does not change significantly. As the heat treatment temperature increased, the atomic diffusion rate accelerated, the grain size increased from 150 to 200 nm, the volume fraction of the BCC phase increased slightly, and the segregation of elements such as Co and Ni became more pronounced in the alloy. The alloy also becomes denser, which reduces the lattice distortion, resulting in a more uniform and orderly porous structure.

The phase structures of the dealloyed samples also changed. At the same dealloying temperature, as the prior heat-treatment temperature increaseda from as-cast to 1000 °C, the diffraction peaks of the FCC and BCC phases gradually broadened, the intensity of the FCC peaks steadily decreased, and the BCC peaks were enhanced. For dealloying at 40 °C, the intensity of the FCC main peak in the as-cast sample was 697, whereas it was only 257 in the sample heat-treated at 1000 °C. For the 60 °C dealloying, the FCC main peak intensity dropped from 525 (as-cast) to 351 (1000 °C heat-treated). For 80 °C dealloying, it decreased from 201 (as-cast) to 119 (1000 °C heat-treated). This is because high-temperature heat treatment accelerates atomic diffusion and phase transformations in the alloy, causing partial dissolution of the FCC phase and its conversion to the BCC phase at high temperatures. At the same heat treatment temperature, the peak intensity of the FCC phase gradually decreased with increasing dealloying temperature. At 40 °C dealloying, only a small amount of the FCC phase dissolved, and the overall phase structure remained similar to that before dealloying. As the dealloying temperature increases to 60 °C and 80 °C, a large fraction of the FCC phase dissolves, its peak intensity progressively weakens, the BCC diffraction peaks become stronger, and the phase fraction is adjusted accordingly.

**Figure 5:**
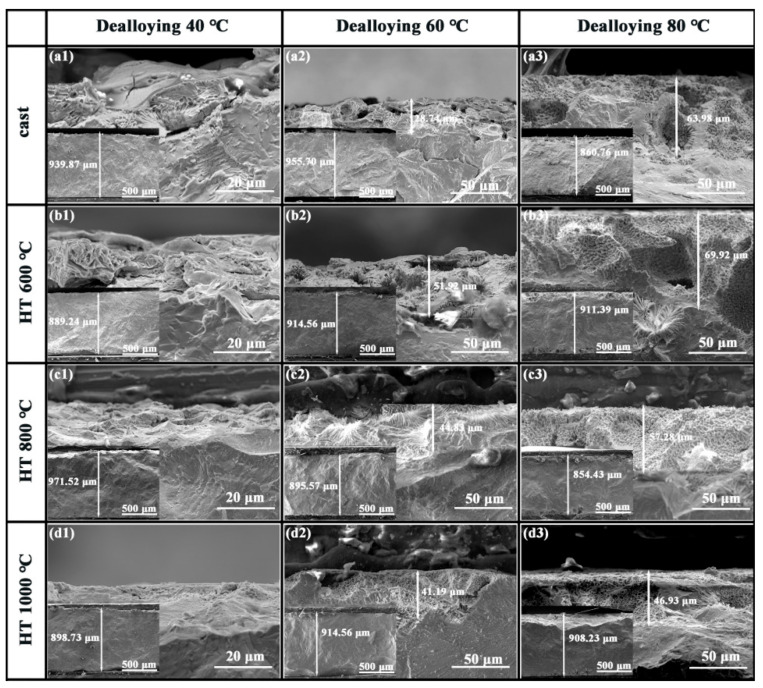
Cross-sectional microstructure (SEM) of CoCrNi0.5Ti0.3V0.2Al0.4 EHEA after heat treatment and dealloying: (**a1**–**d1**) dealloyed at 40 °C; (**a2**–**d2**) dealloyed at 60 °C; (**a3**–**d3**) dealloyed at 80 °C.

Figure 5 shows cross-sectional SEM images of the CoCrNi0.5Ti0.3V0.2Al0.4 EHEA precursor after dealloying at different temperatures. At 40 °C dealloying (Figure 5(a1–d1)), the cross-section exhibits almost no pores. At 60 °C (Figure 5(a2–d2)), the dealloying depth increased, and the as-cast sample exhibited few pores and an uneven pore distribution. With higher prior heat-treatment temperatures, the cross-sectional porous structure and eutectic morphology became more evident. When the dealloying temperature is increased to 80 °C (Figure 5(a3–d3)), the porous layer thickness reaches its maximum and the pore distribution becomes more uniform, indicating that dealloying at 80 °C is more efficient. Simultaneously, as the heat treatment temperature increased, the dealloyed layer thickness decreased, suggesting that heat treatment improved the corrosion resistance of the sample. Overall, increasing the dealloying temperature led to a thicker porous layer, and this uniform porous structure was beneficial for ensuring sufficient contact between the electrolyte and the interior of the alloy, thereby enhancing the efficiency of the electrochemical reaction. Based on these results, the sample dealloyed at 80 °C was selected for electrocatalytic performance testing.

### 3.3. Electrocatalytic Performance

To evaluate the electrocatalytic performance of the prepared porous HEA catalyst, HER and OER measurements were performed in 1 M KOH using a standard three-electrode system. All potentials were calibrated to the reversible hydrogen electrode (RHE) reference.

**Figure 6:**
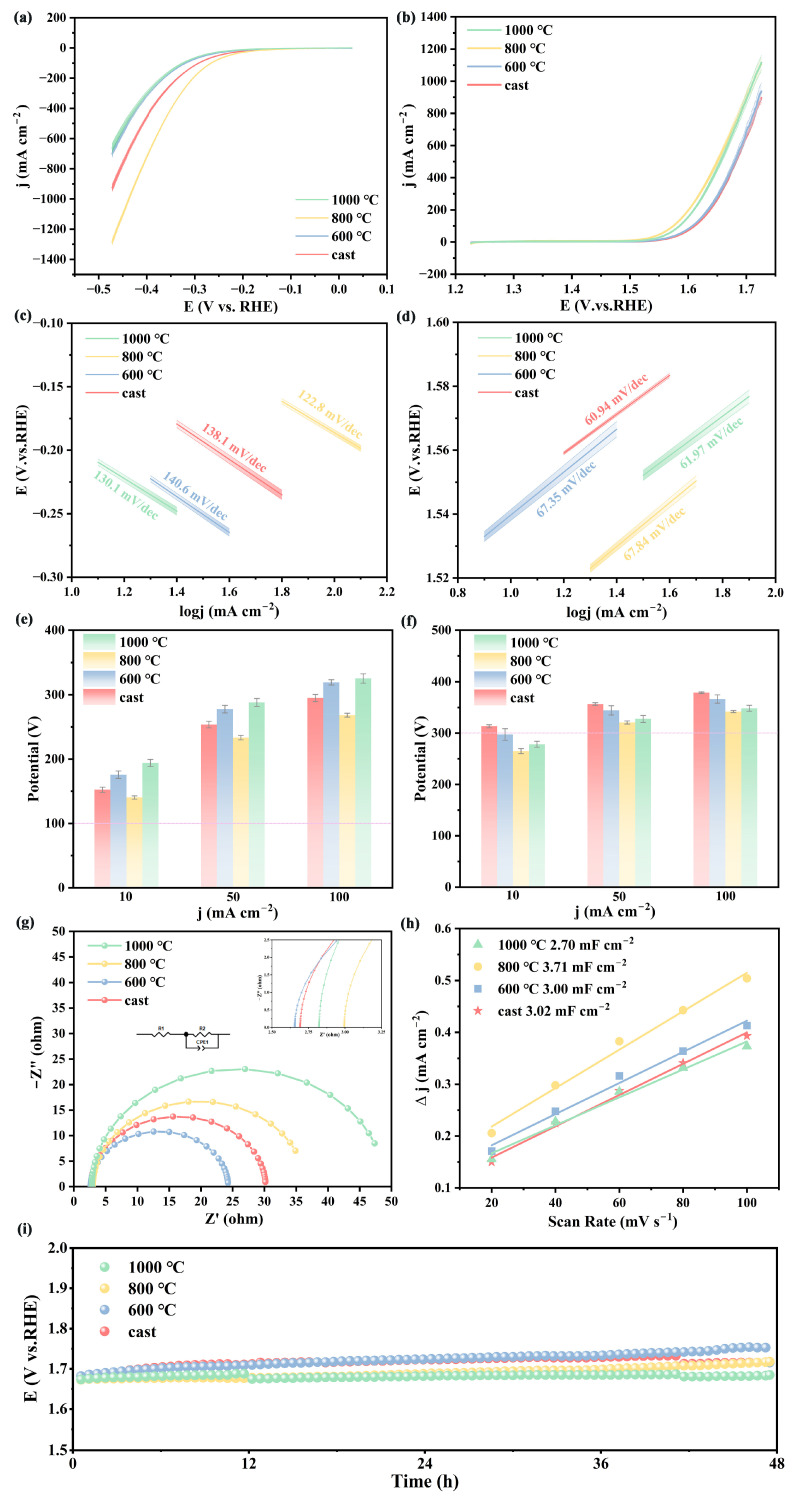
(**a**) LSV polarization curves for HER; (**b**) LSV polarization curves for OER; (**c**) Tafel plots for HER; (**d**) Tafel plots for OER; (**e**) overpotentials at 10 mA·cm^−2^ for HER; (**f**) overpotentials at 10 mA·cm^−2^ for OER; Error bars represent standard deviation from three independent replicates. (**g**) Nyquist plots from EIS (inset: equivalent circuit); (**h**) double-layer capacitance comparison; (**i**) 48 h stability test.

Figure 6a shows the HER polarization curves of the catalysts after different heat treatments. The data error for all samples was less than 5%, indicating that the experimental system had good stability and reproducibility of the results. At a current density of 10 mA·cm^−2^, the HT-800 °C sample exhibited the lowest overpotential of 140 mV, but at 1000 °C, the overpotential increased to 196 mV at the same current density. To meet the completeness requirements of the kinetic analysis, Supplementary Figure S1 shows complete Tafel curves for all the catalysts. The linear fitting regions are marked, and the goodness-of-fit R^2^ values are all higher than 0.995, confirming the reliability of the Tafel slope calculations. Figure 6c shows the Tafel slopes of the catalyst electrodes. The HT-800 °C sample has a Tafel slope of only 122.8 mV/dec, which is much lower than that of the as-cast sample (138.1 mV/dec), the HT-600 °C sample (140.6 mV/dec), and the HT-1000 °C sample (130.1 mV/dec), indicating that the HT-800 °C electrode possesses excellent reaction kinetics. This is because the HT-600 °C sample suffers from elemental segregation and microstructural inhomogeneity, resulting in a smaller electrochemically active surface area, thus limiting the available reactive surface. In contrast, the HT-800 °C treatment exposes a high density of active sites and optimizes the hydrogen adsorption free energy [45], and its porous structure significantly increases the electrochemically active surface area. However, the HT-1000 °C sample experienced severe grain coarsening, a reduction in the active site density, and markedly retarded reaction kinetics.

Figure 6b shows a comparison of the OER polarization curves of the catalysts after different heat treatments. The data error for all samples was less than 5%, indicating that the experimental system had good stability and reproducibility. The as-cast catalyst exhibited relatively poor OER activity, with an overpotential of 314 mV at 10 mA·cm^−2^. At the same current density, the HT-800 °C sample shows the lowest overpotential, only 265 mV, which is lower than that of a commercial RuO_2_ catalyst (290 mV). However, with a further increase in the heat treatment temperature, the overpotential of the HT-1000 °C sample slightly increased to 288 mV at 10 mA·cm^−2^. Figure 6e shows the Tafel slope values for the OER of each catalyst. All samples exhibited low Tafel slopes (<70 mV/dec), with the as-cast sample (60.94 mV/dec), HT-600 °C sample (67.35 mV/dec), HT-800 °C sample (67.84 mV/dec), and HT-1000 °C sample (61.97 mV/dec), indicating that the catalyst electrode exhibited excellent reaction kinetics performance. Although the as-cast sample had the lowest Tafel slope, it exhibited the highest overpotential, which was attributed to the non-uniform microstructure (obvious coarsening of the eutectic region at the grain boundaries) and element segregation, resulting in a limited effective active area. In contrast, the HT-800 °C sample had a slightly higher Tafel slope but exhibited the lowest overpotential, which was attributed to its optimized dual-phase structure and uniform porous morphology, which significantly enhanced the electrochemically active area. Under the same test conditions, the catalyst in this study exhibited performance comparable to that of the NiFe LDH studied by Suliman et al. [46] (Tafel slope of 48.6 mV/dec and overpotential of 270 mV) and slightly outperformed the Co-Mn-P@ Ni-Co nanostructure catalyst (Tafel slope of 56 mV/dec and overpotential of 309 mV) studied by Abedi et al. [47], but its performance was still inferior to that of the Co-Cr-Fe-Ni-Nb high-entropy oxide electrocatalyst (Tafel slope of 42.7 mV/dec and overpotential of 251 mV) studied by Lin et al. This catalyst still has room for improvement in terms of enhancing the kinetic rates and other aspects in the future. The charge transfer resistance (EIS) testing is shown in Figure 6g, where the inset shows the corresponding equivalent circuit diagram. After fitting the Nyquist plots of each catalyst electrode, it was observed that all electrocatalysts had a small solution resistance (Rs) and charge transfer resistance (Rct). The solution resistance used in the test (2.656–2.995 Ω) remained essentially unchanged, indicating that the test environment remained fundamentally stable. The electrochemical impedance spectrum of the HT-600 °C sample exhibited a smaller arc, with a charge transfer resistance of 21.73 Ω, while the charge transfer resistance of the cast catalyst was 27.47 Ω. The charge transfer resistance of the HT-800 °C sample is 38.27 Ω, and the charge transfer resistance of the HT-1000 °C sample is 45.09 Ω, indicating that the HT-600 °C sample has the highest charge transfer efficiency with the electrolyte. To evaluate the electrochemical activity of the samples subjected to different heat treatment temperatures, EIS was fitted to obtain Cdl. The results showed that the HT-800 °C sample had the highest Cdl value of 3.34 mF/cm^2^, while the Cdl values for the cast, HT-600 °C, and HT-1000 °C samples were 2.81 mF/cm^2^, 2.76 mF/cm^2^, and 2.54 mF/cm^2^, respectively. To ensure accuracy, Supplementary Figure S2 shows the multi-scan rate CV curves for each catalyst, with cyclic voltammetry tests conducted at scan rates ranging from 20 to 100 mV/s in the non-Faradaic region. For double-layer capacitance, there is a linear relationship between the current and scan rate: ∆j = (CDL/A) · v + b. The linear slope of the current density data points at each scan rate in the CV cycle represents the double-layer capacitance of the electrode per unit geometric area of the electrode. Therefore, by performing linear fitting with the scan rate (ν) as the x-axis and the current density (Δj) as the y-axis, the slope of the fitted line represents Cdl, as shown in Figure 6h. As shown in the figure, with an increase in the heat treatment temperature, the double-layer capacitance first increased and then decreased, reaching a maximum value (3.71 mF/cm^2^) at HT-800 °C, indicating a larger active area. This is because the optimized microstructure and porous surface are conducive to electron transfer and the adsorption and desorption of intermediate products, thereby accelerating the reaction. The Cdl values obtained from the CV calculations were slightly higher than those obtained from the EIS fitting. This is because the CV method integrates charge at low frequencies, where deep pore responses are primarily observed, capturing a higher capacitive contribution from the overall pore structure of the electrode. In contrast, the EIS method primarily reflects the interface responses at high frequencies and is less sensitive to ion transport in deep pores. Additionally, CV measurements determine the dynamic average capacitance within a wide potential window, whereas EIS characterizes the steady-state instantaneous capacitance at the open-circuit potential. The fundamental methodological differences account for the variations in the Cdl values, but the error is less than 10%, and the performance trends of all catalysts are consistent, indicating that the data possess high reliability. Stability testing (Figure 6i) showed that all samples maintained a current density retention rate of up to 90% after 48 h of constant potential testing, demonstrating excellent electrochemical stability.

As shown in Table 1, the Al-Co-Cr-Fe-Ni EHEA catalyst developed in this study outperforms most advanced materials reported in the literature in terms of overpotential (265 mV) and reaction kinetics (Tafel slope of 67.8 mV/dec). The mechanism behind this performance enhancement can be attributed to the fact that heat treatment optimizes the intrinsic electronic structure of the alloy by regulating the BCC/FCC phase ratio, while dealloying treatment constructs a three-dimensional porous surface through selective corrosion. The synergistic effect of these two processes results in a nanoporous structure that provides abundant active sites, thereby accelerating the reaction kinetics and reducing the activation energy.

### 3.4. Effect of the Electrocatalytic Reaction on the Microstructure of CoCrNi0.5Ti0.3V0.2Al0.4 EHEA

**Figure 7:**
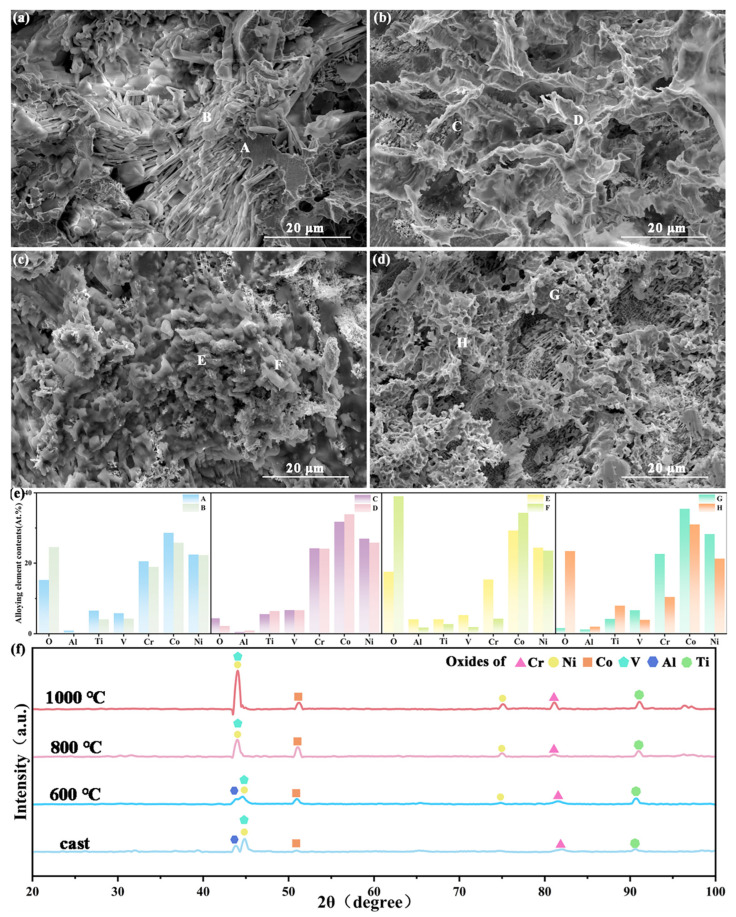
Microstructure, elemental distribution, and phase composition of samples after a 48 h stability test: (**a**–**d**) SEM images of (**a**) as-cast sample, (**b**) 600 °C sample, (**c**) 800 °C sample, (**d**) 1000 °C sample, (**e**) elemental distribution maps, and (**f**) XRD pattern.

After the 48 h constant-potential stability test, the microstructure, elemental distribution, and phase composition of the alloy were examined (Figure 7). The SEM images (Figure 7a–d) reveal that the surface of the as-cast sample has a clearly porous structure with irregular protrusions. As the heat treatment temperature was increased to 800 °C, the surface exhibited more protrusions and pores; at 1000 °C, the number of protrusions decreased. EDS maps (Figure 7e) show that the oxygen content is generally high in all samples owing to the reaction of the alloy with oxygen during the electrolysis process, forming an oxide film on the surface. Notably, the sample heat-treated at 800 °C contained a significantly higher oxygen content, indicating that more intense oxidation occurred during water electrolysis. To further identify the oxide composition, XRD analysis was performed on the samples.

In the as-cast sample after electrolysis, oxides of Ni, Co, V, Ti, and Al were detected. As the heat treatment temperature increased from 600 to 1000 °C, the diffraction peaks of V, Ni, and Co oxides became stronger. The electrocatalytic OER process and long-term stability test induced the in situ formation of a (hydroxy)oxide layer on the alloy surface that was rich in multiple transition metal elements. This complex oxide layer serves as the true active phase; its composition (oxides of Ni, Co, V, and Ti) and synergistic interactions provide optimized active sites. In particular, high-valence metal ions (Ni^3^⁺/^4^⁺, Co^3^⁺/^4^⁺) are key for adsorbing oxygenated intermediates (OH, O, *OOH) and driving the reaction [51], while the presence of oxygen vacancies further adjusts the intermediate adsorption energies and may promote charge transfer [52]. The porous structure significantly increases the electrochemically active surface area, exposing more potential active sites. Its interconnected pore network effectively facilitates the transport of OH^−^ reactants to the active sites and the rapid detachment of O_2_ bubbles, markedly reducing the concentration polarization and bubble coverage effects, thereby sustaining the reaction kinetics at high current densities. The tight integration of the in situ formed, structurally stable porous scaffold with the surface-active oxide layer constructs a highly efficient and durable electrocatalytic interface, enabling the material to maintain high catalytic activity and structural stability during long-term OER operation.

## 4. Conclusions

(1). As the heat treatment temperature increased, the fraction of the FCC phase first increased and then decreased. Heat treatment at 800 °C formed an FCC-dominated dual-phase structure, and heat treatment effectively enhanced the corrosion resistance of the alloy.

(2). Dealloying at 80 °C produces a uniform porous structure with pore sizes of 50–100 μm, which increases the active surface area and significantly improves the electrocatalytic activity and stability of the catalyst.

(3). Electrochemical tests showed that the sample heat-treated at 800 °C achieved an OER overpotential of only 265 mV, exhibiting excellent performance under alkaline conditions.

(4). After a 48 h constant-potential stability test, the surface of the 800 °C sample contained abundant Co and Ni oxides, and the high-valence metal ions effectively promoted oxygen adsorption.

This study provides a novel approach for the microstructural design of high-entropy alloys. The combined processing strategy can be extended to other non-precious metal electrocatalyst systems, promoting the practical application of high-entropy alloys in energy conversion.

## Figures and Tables

**Table 1:** Comparison of alkaline OER catalyst performance.

Catalyst Composition	Electrolyte Type	Overpotentials(mV) 10 mA/cm^2^	Tafel Slopes (mV/dec)
CoCrNi0.5Ti0.3V0.2Al0.4 EHEA	1 M KOH	265	67.84
Ni2Fe1Al97	1 M KOH	244	39
P-Ni30Co30Fe10Cr10Al18W2	1 M KOH	211	41.3
FeCoNiCrZr0.25 HEA	1 M KOH	215	44.9
NiFe LDH	1 M KOH	270	48.6
Co-Mn-P@Ni-Co	1 M KOH	309	56
FeCoMnCuAl HEA [48]	1 M KOH	280	76.13
Co-Cr-Fe-Ni-Nb	1 M KOH	251	42.7
Li-NiFe-LDH/g-C_3_N_4_ [49]	1 M KOH	276	51.1
CoSe_2_@Co_3_O_4_ [50]	1 M KOH	252	69
RuO_2_	1 M KOH	290	82.1

## Data Availability

The original contributions presented in this study are included in the article/Supplementary Materials. Further inquiries can be directed to the corresponding authors.

## Supplementary Materials

The following supporting information can be downloaded at: https://www.mdpi.com/article/10.3390/ma18174015/s1, Figure S1: original complete curves (a) HER; (b) OER; Figure S2: the multi-scan CV curves: (a) as-cast; (b) 600 °C sample; (c) 800 °C sample; and (d) 1000 °C sample.

## References

[1] Hong W.T., Risch M., Stoerzinger K.A., Grimaud A., Suntivich J., Shao-Horn Y. (2015). Toward the Rational Design of Non-Precious Transition Metal Oxides for Oxygen Electrocatalysis. Energy Environ. Sci..

[2] Chen D., Chen C., Baiyee Z.M., Shao Z., Ciucci F. (2015). Nonstoichiometric Oxides as Low-Cost and Highly-Efficient Oxygen Reduction/Evolution Catalysts for Low-Temperature Electrochemical Devices. Chem. Rev..

[3] Lee J.E., Jeon K.-J., Show P.L., Lee I.H., Jung S.-C., Choi Y.J., Rhee G.H., Lin K.-Y.A., Park Y.-K. (2022). Mini Review on H2 Production from Electrochemical Water Splitting According to Special Nanostructured Morphology of Electrocatalysts. Fuel.

[4] Saad A., Liu D., Wu Y., Song Z., Li Y., Najam T., Zong K., Tsiakaras P., Cai X. (2021). Ag Nanoparticles Modified Crumpled Borophene Supported Co_3_O_4_ Catalyst Showing Superior Oxygen Evolution Reaction (OER) Performance. Appl. Catal. B Environ..

[5] Ahmad A., Nairan A., Feng Z., Zheng R., Bai Y., Khan U., Gao J. (2024). Unlocking the Potential of High Entropy Alloys in Electrochemical Water Splitting: A Review. Small.

[6] Wang Z., Liu P., Han J., Cheng C., Ning S., Hirata A., Fujita T., Chen M. (2017). Engineering the Internal Surfaces of Three-Dimensional Nanoporous Catalysts by Surfactant-Modified Dealloying. Nat. Commun..

[7] Li M., Duanmu K., Wan C., Cheng T., Zhang L., Dai S., Chen W., Zhao Z., Li P., Fei H. (2019). Single-Atom Tailoring of Platinum Nanocatalysts for High-Performance Multifunctional Electrocatalysis. Nat. Catal..

[8] Forty A.J. (1979). Corrosion Micromorphology of Noble Metal Alloys and Depletion Gilding. Nature.

[9] Lu Q., Hutchings G.S., Yu W., Zhou Y., Forest R.V., Tao R., Rosen J., Yonemoto B.T., Cao Z., Zheng H. (2015). Highly Porous Non-Precious Bimetallic Electrocatalysts for Efficient Hydrogen Evolution. Nat. Commun..

[10] Dong C., Kou T., Gao H., Peng Z., Zhang Z. (2018). Eutectic-Derived Mesoporous Ni-Fe-O Nanowire Network Catalyzing Oxygen Evolution and Overall Water Splitting. Adv. Energy Mater..

[11] Li M., Lin F., Zhang S., Zhao R., Tao L., Li L., Li J., Zeng L., Luo M., Guo S. (2024). High-Entropy Alloy Electrocatalysts Go to (Sub-)Nanoscale. Sci. Adv..

[12] Yeh J.W., Chen S.K., Lin S.J., Gan J.Y., Chin T.S., Shun T.T., Tsau C.H., Chang S.Y. (2004). Nanostructured High-Entropy Alloys with Multiple Principal Elements: Novel Alloy Design Concepts and Outcomes. Adv. Eng. Mater..

[13] Cantor B., Chang I.T.H., Knight P., Vincent A.J.B. (2004). Microstructural Development in Equiatomic Multicomponent Alloys. Mater. Sci. Eng. A-Struct. Mater. Prop. Microstruct. Process..

[14] Miracle D.B., Senkov O.N. (2017). A Critical Review of High Entropy Alloys and Related Concepts. Acta Mater..

[15] Zhang Y., Zuo T.T., Tang Z., Gao M.C., Dahmen K.A., Liaw P.K., Lu Z.P. (2014). Microstructures and properties of high-entropy alloys. Prog. Mater. Sci..

[16] Zhen P., Jian S., Luan H.W., Na C., Yao K.F. (2023). Effect of Mo on the high temperature oxidation behavior of Al_19_Fe_20−x_Co_20−x_Ni_41_Mo_2x_ high entropy alloys. Intermetallics.

[17] Zhu H., Zhu Z., Hao J., Sun S., Lu S., Wang C., Ma P., Dong W., Du M. (2022). High-Entropy Alloy Stabilized Active Ir for Highly Efficient Acidic Oxygen Evolution. Chem. Eng. J..

[18] Louis M.P., Peter F. (1979). A synthesis of the Brewer-Engel and Samsonov-Pryadko-Pryadko electron correlations for metals. J. Solid State Chem..

[19] Lin Y., Xu W., Gao Z., Liang Y., Jiang H., Li Z., Wu S., Cui Z., Sun H., Zhang H. (2024). Self-Supporting High-Entropy Co-Cr-Fe-Ni-Nb Oxide Electrocatalyst with Nanoporous Structure for Oxygen Evolution Reaction. Chem. Eng. J..

[20] Chongjun Z., Wenlei C., Nan S., Shi C., Wenbin J., Chunhua Z. (2024). Facile preparation of porous high-entropy alloy FeCoNiCuMn and its OER performance. J. Phys. Chem. Solids.

[21] Zhu J., Bian P., Sun G., Zhang J., Lou G., Song X., Zhao R., Liu J., Xu N., Li A. (2025). Practical High-Voltage Lithium Metal Batteries Enabled by the In-Situ Fabrication of Main-Chain Fluorinated Polymer Electrolytes. Angew. Chem. Int. Ed..

[22] Wang C., Zhao S., Han G., Bian H., Zhao X., Wang L., Xie G. (2024). Hierarchical Porous Nonprecious High-entropy Alloys for Ultralow Overpotential in Hydrogen Evolution Reaction. Small Methods.

[23] Huang K., Xia J., Lu Y., Zhang B., Shi W., Cao X., Zhang X., Woods L.M., Han C., Chen C. (2023). Self-Reconstructed Spinel Surface Structure Enabling the Long-Term Stable Hydrogen Evolution Reaction/Oxygen Evolution Reaction Efficiency of FeCoNiRu High-Entropy Alloyed Electrocatalyst. Adv. Sci..

[24] Lu F., Zong L., Zhang G., Li P., Fan K., Jiang S., Chen X., Liu P., Wang L. (2025). Ultrafine high entropy alloys with Ru activated sites for highly durable and industrial grade electrocatalytic water splitting. Compos. Part B Eng..

[25] Lu Y., Dong Y., Guo S., Jiang L., Kang H., Wang T., Wen B., Wang Z., Jie J., Cao Z. (2014). A Promising New Class of High-Temperature Alloys: Eutectic High-Entropy Alloys. Sci. Rep..

[26] Shi H., Zhou Y.T., Yao R.Q., Wan W.B., Zhang Q.H., Gu L., Wen Z., Lang X.Y., Jiang Q. (2020). Intermetallic Cu5Zr Clusters Anchored on Hierarchical Nanoporous Copper as Efficient Catalysts for Hydrogen Evolution Reaction. Research.

[27] Luo M., Peng W., Zhao Y., Lan J., Peng M., Han J., Li H., Tan Y. (2021). Dilute molybdenum atoms embedded in hierarchical nanoporous copper accelerate the hydrogen evolution reaction. Scr. Mater..

[28] Chen Q., Han X., Xu Z., Chen Q., Wu Q., Zheng T., Wang P., Wang Z., Wang J., Li H. (2023). Atomic phosphorus induces tunable lattice strain in high entropy alloys and boosts alkaline water splitting. Nano Energy..

[29] Zhang Q., Guo Q., Zhang Y., He Y., Gong W., Liu W., Liu X., Li R. (2025). Architecting Gradient Hierarchically Porous Catalyst via Negative Mixing Enthalpy High-Entropy Alloy for Durable Water Splitting at Ampere-Level Current Density. Adv. Funct. Mater..

[30] Yao R.Q., Zhou Y.T., Shi H., Wan W.B., Zhang Q.H., Gu L., Zhu Y.F., Wen Z., Lang X.Y., Jiang Q. (2021). Nanoporous Surface High-Entropy Alloys as Highly Efficient Multisite Electrocatalysts for Nonacidic Hydrogen Evolution Reaction. Adv. Funct. Mater..

[31] Li R., Liu X.J., Wang H., Zhou D.Q., Wu Y., Lu Z.P. (2016). Formation mechanism and characterization of nanoporous silver with tunable porosity and promising capacitive performance by chemical dealloying of glassy precursor. Acta Mater..

[32] Munitz A., Salhov S., Hayun S., Frage N. (2016). Heat treatment impacts the micro-structure and mechanical properties of AlCoCrFeNi high entropy alloy. J. Alloys Compd..

[33] Zahra Z., Milad Z., Amir M., Saeed S., Mahesh S. (2023). Effect of heat treatment regime on microstructure and phase evolution of AlMo0.5NbTa0.5TiZr refractory high entropy alloy. J. Alloys Compd..

[34] Li Y., Yang Z., Duan H., Ma Z., Wu C., Bai Y., Sun C., Wang P., Li J. (2023). Microstructure and mechanical properties of Al0.4Co0.5V0.6FeNi high-entropy alloys processed by homogenization treatment. Intermetallics.

[35] Sun H., Liu T., Hashimoto N., Oka H. (2024). Effects of vacuum heat treatment on novel Co-free Al_0.9_Cr_0.8_FeMn_0.8_Ni_2.0_ eutectic high entropy alloy: Microstructure evolution and mechanical properties. Vacuum.

[36] Zhang G., Ming K., Kang J., Huang Q., Zhang Z., Zheng X., Bi X. (2018). High entropy alloy as a highly active and stable electrocatalyst for hydrogen evolution reaction. Electrochim. Acta.

[37] Gao Y., Han J., Ge F., Zhang X., Cai Y., Cui Y. (2024). Design of eutectic high-entropy alloys in the Co-Cr-Ni-V-Ti-Al system using Scheil solidification path optimization. J. Alloys Compd..

[38] Chen X., Xie W., Zhu J., Wang Z., Wang Y., Ma Y., Yang M., Jiang W., Yu H., Wu Y. (2021). Influences of Ti additions on the microstructure and tensile properties of AlCoCrFeNi2.1 eutectic high entropy alloy. Intermetallics.

[39] He J.Y., Liu W.H., Wang H., Wu Y., Liu X.J., Nieh T.G., Lu Z.P. (2014). Effects of Al addition on structural evolution and tensile properties of the FeCoNiCrMn high-entropy alloy system. Acta Mater..

[40] Yan P.X., Chang J., Wang W.L., Zhu X.N., Lin M.J., Wei B.J.A.M. (2022). Eutectic growth kinetics and microscopic mechanical properties of rapidly solidified CoCrFeNiMo0.8 high entropy alloy. Acta Mater..

[41] Kyoungdoc K., Peter W.V. (2018). Ostwald ripening of spheroidal particles in multicomponent alloys. Acta Mater..

[42] Zhao L., Zhisong C., Lingyu W., Zhou W., Qi L., Jianfeng W., Wei X. (2024). Effect of Cr on high-temperature oxidation resistance of Cr–Si–Mn alloyed press-hardened steel during press hardening. J. Mater. Res. Technol..

[43] Zhang Y., Li J., Wang J., Wang W.Y., Kou H., Beaugnon E. (2018). Temperature dependent deformation mechanisms of Al_0.3_CoCrFeNi high-entropy alloy, starting from serrated flow behavior. J. Alloys Compd..

[44] Song L., Hu W., Liao B., Wan S., Guo X. (2023). Corrosion behavior of AlCoCrFeNi_2.1_ eutectic high-entropy alloy in Cl--containing solution. J. Alloys Compd..

[45] Qi L., Guan J. (2025). Electronic structure modulation of high entropy materials for advanced electrocatalysis. Green Energy Environ..

[46] Munzir S., Abdullah A.G., Turki B.Q.D., Mohd R.Z.Y., Mohammad Q. (2022). Growth of ultrathin nanosheets of nickel iron layered double hydroxide for the oxygen evolution reaction. Int. J. Hydrogen Energy.

[47] Rokhsareh A., Ghasem B.D. (2025). Interfacial surface engineering of Co-Mn-P ultrathin nanosheets on Ni-Co hierarchical nanostructure for boosting electrochemical active sites in overall water splitting. J. Power Sources.

[48] Zhu X., Huang W., Lou Y., Yao Z., Ying H., Dong M., Tan L., Zeng J., Ji H., Zhu H. (2024). Ultrafast joule-heating synthesis of FeCoMnCuAl high-entropy-alloy nanoparticles as efficient OER electrocatalysts. Prog. Nat. Sci. Mater. Int..

[49] Li Z., Liu M., Yan J., Lee L.Y.S. (2023). A “doping–interfacing” Strategy Enables Efficient Alkaline Freshwater and Seawater Oxidation by NiFe-layered Double Hydroxides. Chem. Eng. J..

[50] Abdul H., Muhammad Y.S., Muhammad N.L., Abdulaziz A., Muhammad A.S., Abdul J.L., Imtiaz A.S., Muhammad I.A., Mukesh K., Umair A. (2024). CoSe_2_@Co_3_O_4_ nanostructures: A promising catalyst for oxygen evolution reaction in alkaline media. Catal. Commun..

[51] Frydendal R., Paoli E.A., Knudsen B.P., Wickman B., Malacrida P., Stephens I.E.L., Chorkendorff I. (2014). Benchmarking the Stability of Oxygen Evolution Reaction Catalysts: The Importance of Monitoring Mass Losses. ChemElectroChem.

[52] Cheng F., Zhang T., Zhang Y., Du J., Han X., Chen J. (2013). Enhancing Electrocatalytic Oxygen Reduction on MnO_2_ with Vacancies. Angew. Chem. Int. Ed..

