# Supplementary material for: Heat Treatment-Assisted Optimization of the Water Splitting Performance of CoCrNi0.5Ti0.3V0.2Al0.4 Eutectic High-Entropy Alloy
Source: Materials (Basel). 2025 Aug 27;18(17):4015. doi: 10.3390/ma18174015 (PMC12428961; doi:10.3390/ma18174015)
Supplement: Supplementary file 1 [file materials-18-04015-s001.zip › materials-3775878-supplementary.pdf]

Supporting Information for

Heat Treatment-Assisted Optimization of the Water Splitting Performance of CoCrNi0.5Ti0.3V0.2Al0.4 Eutectic High-Entropy Alloy

Mingran Sun [1,2], Zixiang Yin [1,2], Shuai Liu [1,2], Yangchuan Cai [1,2,*] and Yu Zhang [1,2,*]

1 School of Materials Science and Engineering, Tianjin University of Technology, Tianjin 300384, China; m15081643686@163.com (M.S.); 15290259270@163.com (Z.Y.); 15933687828@163.com (S.L.)

2 Tianjin Key Laboratory of Advanced Functional Porous Materials, Institute for New Energy Materials and Low-Carbon Technologies, School of Materials Science and Engineering, Tianjin University of Technology, Tianjin 300384, China

* Correspondence: 18622265848@163.com (Y.C.); yzhang8@email.tjut.edu.cn (Y.Z.); Tel: +86-18622265848 (Y.C.)

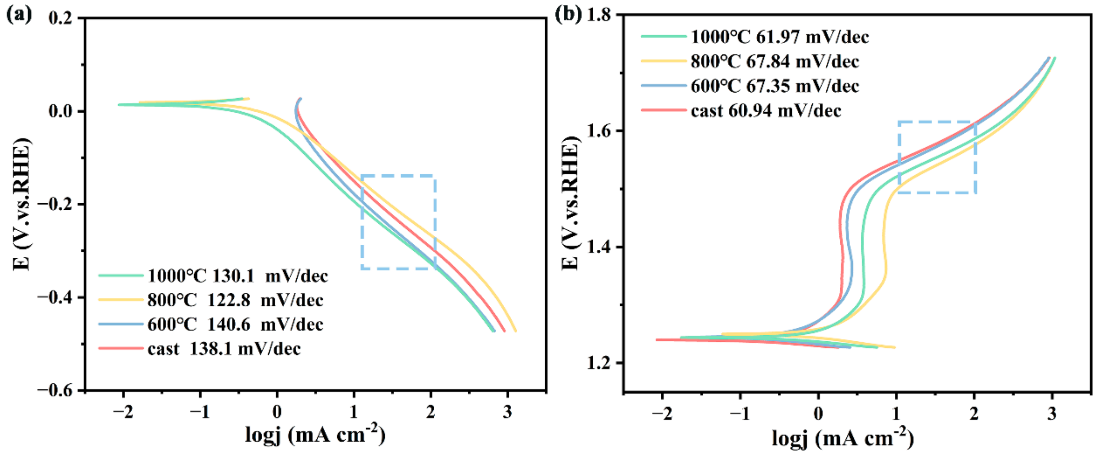

Supplementary Figure S1: original complete curves (a) HER; (b) OER.

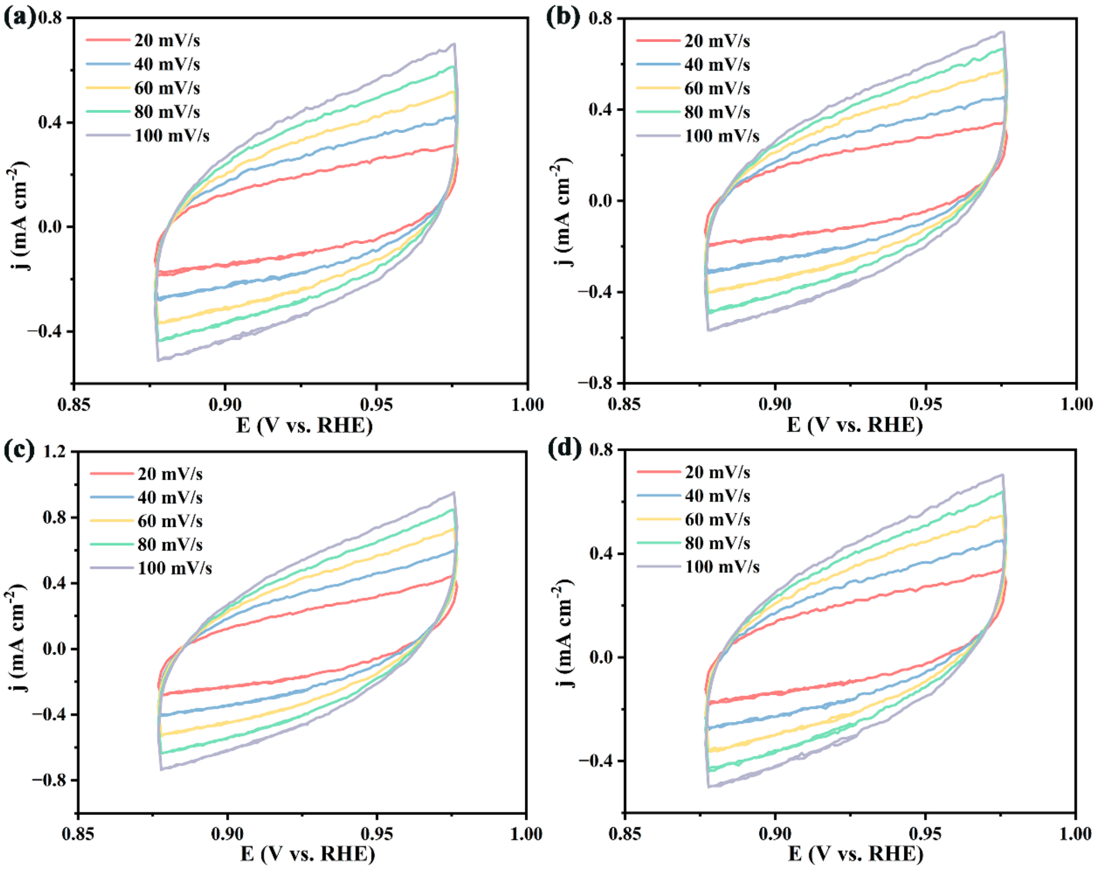

Supplementary Figure S2: the multi-scan CV curves : (a) as-cast; (b) 600 °C sample; (c) 800 °C sample; and (d) 1000 °C sample.
